# Impact of freezing rate on electrical conductivity of produce

**DOI:** 10.1186/2193-1801-2-633

**Published:** 2013-11-25

**Authors:** Francesco Marra

**Affiliations:** Dipartimento di Ingegneria Industriale, Università degli studi di Salerno, Fisciano, SA Italy

**Keywords:** Ohmic processing, Texture, Electrical conductivity, Electro-heating, Food, Frozen

## Abstract

The aim of this work was to compare the effects of freezing rate on electrical conductivity of potatoes, carrots and apples. Electrical conductivity tests were conducted on a custom ohmic cell while samples texture was measured by means of a universal testing machine. The raw un-pretreated samples were used as control. This study showed that freezing pre-treatments lead to differences in electrical conductivity of considered samples, producing structural damage, the latter being relatively more severe when the tested products undergo ohmic treatment.

## Findings

### Background

While microwave (MW) heating has become popular in both domestic and industrial applications, other electro-heating applications, such as radio frequency (RF) and ohmic (OH) heating are gaining importance in the industrial and scientific community, since they showed to be applicable to a wide of processes, from cooking to thawing (Farag *et al*., 2010), to sterilization (Sun *et al*., 2008), improving product quality and reducing processing times (McKenna *et al*., 2006, Somavat *et al*., 2012).

The main advantages of OH processing are the rapid and relatively uniform heating achieved by means of the direct passage of electric current through the product. In addition, processing times are substantially reduced in relation to conventional heating which results in higher product quality particularly with respect to product integrity, flavor and nutrient retention (Ozkan *et al*., 2004; Shirsat *et al*., 2004).

Other studies conducted in the field of electrical (OH) and dielectric (RF, MW) heating of foods demonstrated that many processing factors influence the heating (Romano and Marra, 2008; Wang and Sastry, 1993, 2000) but also that composition, pretreatments and storage conditions in frozen chain may alter the properties of the product under processing (Lyng *et al*., 2013; Sarang *et al*., 2007). As stated by Zaritzky ( 2000), freezing operations can have marked effects on the structure of foods at a cellular level and freezing rate influences the potential magnitude of cellular disruption, from a low to a high level. During slow freezing conditions, there is a tendency for a small number of relatively large ice crystals to form in the extracellular space, with a consequent major disruptive effect on cellular structure (Farag *et al*., 2009). On the other hand, fast freezing leads to the formation of a large number of relatively small ice crystals, which form within and between cells. These small crystals have a much less disruptive effect on the cellular structure of foods (Lyng *et al*., 2013).

The aim of this work was to verify the effects of freezing pretreatment on electric conductivity of fresh solid food products (potatoes, carrots and apple), subjected to constant electrical field strength.

## Materials and methods

### Raw material

Three types of foods were chosen in this work: potatoes and carrots, as typical vegetables used as basis of soups or to be consumed a side of main dishes; apples as one of most diffused fruits, easy to find on the market and, once processed, to be used as fruit-in-syrup or as ingredient for other food preparations. In details, potatoes (*Solanum tuberosum L*.) of Arielle variety, carrots (*Daucus carota* var. *sativus*) of Flakkee extra variety, and apples (*Malus domestic*) of Golden Delicious variety were bought in a local market and were stored in a ventilated cooled room, at a temperature between 8°C and 14°C.

For each product and for each OH treatment, ten cylinders (9 mm height, 30 mm diameter) of unfrozen controls were prepared, using a circular cutter made on purpose. Totally, ninety cylinders of unfrozen controls were prepared.

### Freezing and thawing procedures

In order to examine the effect of freezing rate on the electric conductivity of considered products, entire vegetables and fruits were prepared that were either frozen slowly by placing in a cold storage room at -18°C for two days or rapidly frozen by immersion in liquid nitrogen for 10 minutes and subsequently were placed in a cold storage room at -18°C for two days.

Prior to the measurement of electrical conductivity foods were defrosted in a chill at 4°C, then samples were shaped (by means of the same circular cutter used for preparing the unfrozen controls) as cylinders (9 mm height, 30 mm diameter) obtained of the inner part of the foods just before undergoing the experiments in a OH cell, as below described, and subsequently equilibrated in an air conditioned laboratory (25°C). For each product and for each OH treatment, twenty frozen samples were prepared. Totally, hundred eighty frozen samples were prepared.

### Measurement of electrical conductivity

The OH cell used in this work to measure the electrical conductivity is the one described into details by (.,Olivera *et al*^2013^), working at 50 Hz and imposing an electric field strength of 3300 V/m.

Electrical conductivity (*σ*) was calculated according using the following equation:
1

where *I* is the current intensity (measured in A), *V* is the voltage (V), *L* is the gap between the electrodes (m) and *A* is the electrode surface area (m^2^).

 (Olivera *et al.*^2013^) demonstrated that the three products considered in this paper show a relatively low heating rate if submitted to an average electric field strength of 3300 V/m. In any case, in order to monitor the temperature during the measurement of electrical conductivity, K-thermocouples (Tersid, Italy) were inserted into the sample’s core and hold in it. Electrical conductivity of samples has been measured at 25°C, 37°C and 50°C.

### Texture measurement

According with the procedure presented by Olivera et al., ( 2013), tissue damage degree was estimated from the firmness disintegration index *Z*, defined as in the following equation:
2

where *F(t)* is the measured firmness in N/mm; *F*_*i*_ is the firmness of intact tissue (raw); and *F*_*∞*_ is the firmness of totally destroyed tissue. Conventional (in boiling water for 15 minutes) cooked tissues were used for the determination of the firmness of totally destroyed tissue *F*_*∞*_.

Firmness was defined as the force (measured in N) to deformation (in mm) ratio from the steep linear portion of the compression curve obtained by using a universal testing machine Instron 4301 (Instron Inc, Canton, MA), using a 100 N load cell. Uniaxial compression analysis was performed, at room temperature (~25°C). Samples were compressed (65% compression) on a non-lubricated platform using a flat disk probe, with a constant crosshead speed of 20 mm/min. The raw untreated sample was used as control. Ten replicate experiments were conducted and data were statistically analyzed (α = 0.05).

### Statistical analysis

A one-way analysis of variance (ANOVA) was conducted using Matlab (The Mathworks, MA, USA).

## Results and discussion

**Figure 1:**
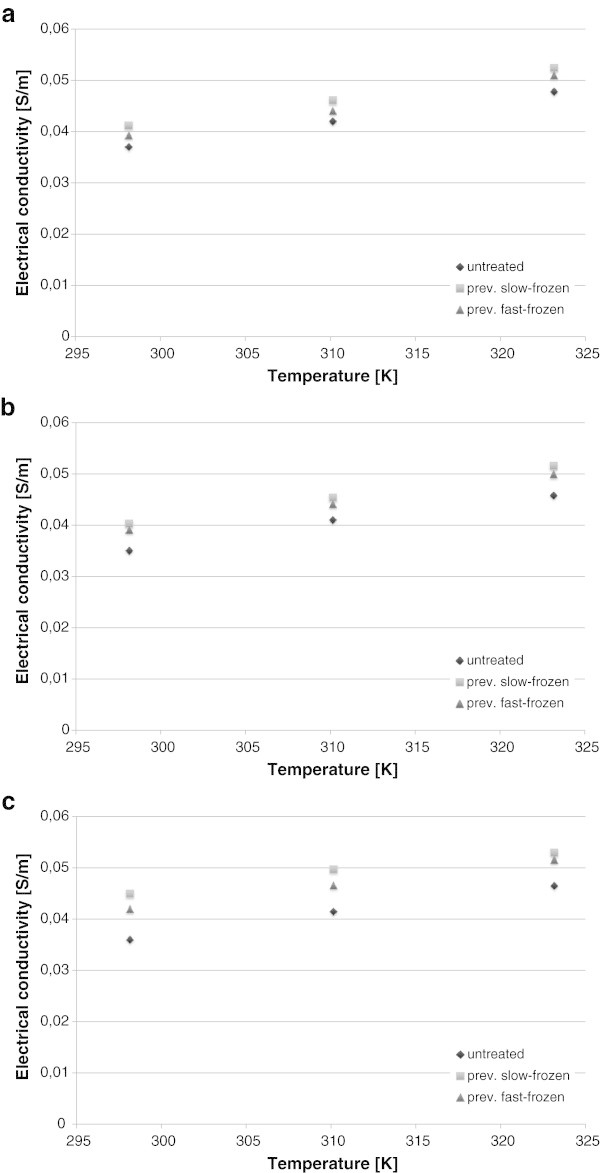
**Electrical conductivity (taken at 50 Hz, 3300 V/m) of: a) potato; b) carrot; c) apple.**

Comparison of electrical conductivity values measured for the three food samples is shown in Figure 1, where measured values of electrical conductivity are plotted as a function of the treatment (untreated, slow frozen, fast frozen) and of sample temperature. All samples, in all the investigated cases, exhibited an electrical conductivity below 0.1 S/m: this is consistent with other values available in literature (Castro *et al*., 2003.,, Olivera *et al*^2013^) as no pretreatments in brine solution were done. As previously observed by (.,Olivera *et al*^2013^), differences among the three samples (control) are confined in 10^-2^ S/m. The same statement remains valid when samples subjected to freezing are considered. When samples were untreated, potato exhibited the higher values of electrical conductivity, followed by the values measured for apple and then for carrot. When samples were previously frozen, apple exhibited the higher values of electrical conductivity. Overall, previously slow frozen samples resulted in slightly higher values of electrical conductivity. Since the overall composition of samples did not change during the freezing and defrosting processes, changes in electrical conductivity were due to changes in the structure of the foods themselves, as addressed later.

Samples of the three chosen foods underwent OH treatment, at an electric field strength of 3300 V/m. The OH process was stopped after 120 and 240 seconds and samples underwent measurement of their firmness. Unfrozen and untreated samples were chosen as control: among them, carrot exhibited the higher firmness (32.41 N/mm), followed by the potato (18.17 N/mm) and then by the apple (14.31 N/mm). Firmness of previously frozen samples was measured before and after the OH. Results are reported in Tables 1, 2 and 3, respectively for potato, carrot and apple.

**Table 1:** **Firmness of potato samples**

	Control (Unfrozen)		Previously slow frozen		Previously fast frozen	
Duration of OH treatment [s]	Firmness [N/mm]					
	Value	S.D.	Value	S.D.	Value	S.D.
0	18.17	0.95	15.73	0.77	16.28	0.76
120	16.67	0.82	14.44	0.76	15.12	0.78
240	14.99	0.61	12.86	0.55	13.49	0.65

**Table 2:** **Firmness of carrot samples**

	Control (Unfrozen)		Previously slow frozen		Previously fast frozen	
Duration of OH treatment [s]	Firmness [N/mm]					
	Value	S.D.	Value	S.D.	Value	S.D.
0	32.41	1.67	29.34	1.65	30.11	1.55
120	27.79	1.63	24.69	1.19	25.92	1.36
240	25.12	1.24	21.04	1.11	22.35	1.17

**Table 3:** **Firmness of apple samples**

	Control (Unfrozen)		Previously slow frozen		Previously fast frozen	
Duration of OH treatment [s]	Firmness [N/mm]					
	Value	S.D.	Value	S.D.	Value	S.D.
0	14.31	0.88	12.73	0.63	13.08	0.69
120	11.34	0.51	10.05	0.49	10.92	0.64
240	8.93	0.48	7.75	0.45	8.21	0.49

As shown in Tables 1, 2, 3, for an electric field strength of 3300 V/m, while the OH process went on, the firmness of all the considered samples decreased, with a slope slightly more pronounced for apple samples (P < 0.05). Final firmness of unfrozen carrot was the highest (25.12 N/mm), followed by the potato (14.99 N/mm) and then by the apple (8.93 N/mm). For all the considered foods, the previously slow-frozen samples resulted in lowest firmness, at any OH processing times.

The firmness disintegration index for each of the samples was computed considering the firmness of untreated samples at time zero as reference values. Results are reported in Tables 4, 5 and 6, respectively for potato, carrot and apple. Before OH treatment, all samples showed an increased firmness disintegration index. Particularly, potato exhibited the higher structural damage, both for previously slow frozen (21.09%) and fast frozen samples (16.34%), while the indexes of firmness disintegration for carrot and apple samples were respectively 13.70% and 13.97% (for previously slow frozen ones) and 10.26% and 10.88% (for previously fast frozen ones). When OH treatment was applied, apple showed the higher structural damage, while potato shown the lower structure damage, in all considered cases. The freezing rate had a clear influence on the firmness disintegration index of all the samples, the slow frozen ones being more sensitive to structure damage with respect to fast frozen ones. The combination of freezing - both at slow and faster rates – with the passage of electrical current has induced a damage on the cellular structure of the considered foods.

**Table 4:** **Firmness disintegration index (%) of potato samples**

	Control (Unfrozen)	Previously slow frozen	Previously fast frozen
Duration of OH treatment [s]	Firmness disintegration index (%)		
0	0	21.09	16.34
120	12.69	32.24	26.36
240	27.48	45.89	40.45

**Table 5:** **Firmness disintegration index (%) of carrot samples**

	Control (Unfrozen)	Previously slow frozen	Previously fast frozen
Duration of OH treatment [s]	Firmness disintegration index (%)		
0	0	13.70	10.26
120	20.62	34.45	28.96
240	32.53	50.74	44.89

**Table 6:** **Firmness disintegration index (%) of apple samples**

	Control (Unfrozen)	Previously slow frozen	Previously fast frozen
Duration of OH treatment [s]	Firmness disintegration index (%)		
0	0	13.97	10.88
120	26.26	37.67	29.97
240	47.57	58.00	53.93

The differences observed in the three analyzed foods are similar to those reported by (,Olivera *et al.*^2013^) and by (.,Lebovka *et al*^2005^) who explained the behavior of the analyzed foods in terms of differences in tissue structure, size of cells, and content of air cavities. According to (,Luo *et al.*^1992^), softening is due largely to the breakdown of pectin but also of other cell walls constituents, such as cellulose and hemicelluloses. The apple structure is rich in pectin, that allow the maintain the cellular structure and this explains why all the treated apple samples exhibited a more sever structural damage. The carrot structure is divided into xylem (that is typically made by hard wall cells) and phloem (made by relatively soft-walled cells), for which the OH can cause dissolution of cell wall components and dissolution of protopectin and, thus, softening; the softening is then accelerated by the freezing treatment (at slower or faster rate). The potato structure is characterized by a cellular array presenting smaller cells at the inner core and larger ones in the outer core, all – independently by their position – of same shape (,Konstankiewicz *et al.*^2002^); OH and freezing treatments both cause the walls breakdown of large cells, thus accelerating the softening.

## Conclusions

Potato, carrot and apple before and after freezing and defrosting exhibited low electrical conductivity. In any case, higher electrical conductivity was measured after products were frozen and then defrosted. Products undergoing slow freezing exhibited different values of their electrical conductivity compared to fast frozen ones.

## Abbreviations

OH: Ohmic
MW: Microwave
RF: Radio-frequency
S.D.: Standard deviation.
